# Why Climate Action Is Global Health Action

**DOI:** 10.4269/ajtmh.22-0189

**Published:** 2022-07-18

**Authors:** American Society of Tropical Medicine and Hygiene (ASTMH) Green Task Force Members

## Abstract

The impacts of climate change on global health and populations are far-reaching, yet they disproportionately affect vulnerable groups, thereby exacerbating disparities. As humanity reckons with the emergency of climate change, our global health community needs to contend with our own contributions to greenhouse gas emissions. We know that transformation is possible and that climate action is the antidote to the existential challenge. As a global health community, we have an immense opportunity, responsibility, and commitment to lead, support, inspire, and empower climate action, research, and innovation that align deeply with our mission and core values.

## THE IMPACTS OF CLIMATE CHANGE

The average global surface temperature was 1.09°C higher (observed range: 0.95–1.20) in 2011–2020 than in preindustrial times.^1^ This increase is causing extreme weather that threatens human life and human health.^2^ The physical impacts of climate change, such as extreme heat events, worsen with increasing temperatures. At present, extreme heat events are occurring 4.8 times (range: 2.3–6.4) more commonly than in preindustrial times.^1^ It is predicted that, in the next 20 years, the average global surface temperature will reach or exceed 1.5°C of warming compared with preindustrial levels, and, of concern, the likelihood of extreme heat events, compared with preindustrial rates, is predicted to increase by 8.6 times (range: 4.3–10.7 times) with an average temperature increase of 1.5°C and 13.9 times (range 6.9–16.6 times), with an average temperature increase of 2°C.^3^

Climate change impacts, such as extreme heat, contribute to myriad health problems including dehydration, pregnancy complications, infectious diseases, and cardiorespiratory morbidity and mortality.^4^ The physical effects of climate change such as flooding, droughts, and fires threaten lives and communities directly and can also cause human conflicts over increased scarcity of resources and/or population displacement. Displaced populations are often at greater risk of poor health as well as poor access to health care services, creating humanitarian crises.^5^ Climate change–related damage to ecosystems coupled with declining biodiversity have been linked to emerging infectious diseases.^6^ Changes in temperature, humidity, rainfall, and land use affect the transmission of vector-borne diseases.^7^^–^^9^ Greenhouse gas emissions, particulates, waste from the fossil fuel industry, and vehicle exhausts contribute to pulmonary and other diseases.^10^ Climate change impacts have detrimental effects on mental health, including increased anxiety, depression, acute stress, and posttraumatic stress disorder (PTSD).^2^^,^^11^

Some people are harmed by climate change more than others, and as a result, climate change is exacerbating inequalities around the world (Box 1).^2^ Health impacts of climate change disproportionately affect those with underlying vulnerabilities, including pregnant women, the elderly, and people with comorbidities, and communities underserved by the health system including mobile groups and migrant workers. Children and adolescents are at particular risk of increased anxiety, depression, acute stress, and PTSD associated with climate change.^2^^,^^11^ Communities most at risk of negative health impacts caused by air pollution are those living or working closest to congested roads, fossil fuel industrial sites, and incinerators. In the United States, African Americans, people of Latin American heritage, and young people are disproportionately exposed to poor air quality.^10^ In low and middle income countries (LMICs) people’s health is also often at risk from burning fuels indoors. Economic hardship exacerbated by climate change is most likely to affect people who are already poor and marginalized or who rely on economies that are threatened, such as smallholder farmers. Disruptions in the aftermath of extreme weather events are associated with increased violence against women, girls, and other vulnerable people.^2^ Additionally, climate change disproportionately disadvantages young people; it has been predicted that children born in 2020 will experience 2- to 7-fold more extreme weather events than those born in 1960.^12^

## OUR OWN EMISSIONS ARE HARMING GLOBAL HEALTH

The activities and operations of global health institutions generate greenhouse gas emissions and contribute to global warming. In our field, greenhouse gas emissions come from energy used to power laboratories, clinics, data centers, animal facilities, and other buildings; fuel burned during commuting and air travel; embodied carbon in equipment and consumables; and emissions supported by financial holdings associated with the fossil fuel industry. Health care accounts for approximately 5% of national carbon footprints.^13^ In 2019, the total greenhouse gas emissions associated with air travel to the American Society of Tropical Medicine and Hygiene (ASTMH) annual meeting was an estimated 8,646 metric tons of carbon dioxide equivalents, which is approximately the same amount of carbon dioxide released from burning 20,017 barrels of oil.^14^^,^^15^ In comparison, the emissions associated with the virtual conference in 2021 was approximately 1.3 metric tons or 3 barrels of oil.^16^

## THE BENEFITS OF ACTION

Reducing our carbon emissions aligns with our mission and core values to improve global health and equality. There are also further benefits. Reducing air pollution improves the respiratory health of local populations. Preserving biodiversity and environmental ecology mitigates against risks of emerging infectious diseases and future pandemics.^17^ Decarbonization to limit warming to no greater than 1.5°C above preindustrial levels prevents even more dangerous climate change, even worse health impacts, and increased inequality. We need to embark on a collective effort to rapidly and significantly reduce these emissions.

## WHAT ARE THE OPPORTUNITIES FOR THE GLOBAL HEALTH COMMUNITY?

There are opportunities for climate action for all global health organizations. The actions we take should be guided by the principles of climate justice and a “just transition.” Historically, high-income countries (HICs) have made the largest contribution of greenhouse gasses to the atmosphere and as such need to rapidly decarbonize to help mitigate the worst effects of climate change. LMICs have contributed less and are often more at risk, but while they may still rely heavily on fossil fuels as a source of energy, they should not be left behind in the global transition to clean energy and environmentally sustainable economies.^18^

Institutions need to develop ambitious decarbonization goals and implementation plans that reduce emissions used to power buildings and transportation, embodied carbon in equipment and consumables, and financial investments in the fossil fuel industry. Decarbonization goals should align with the latest evidence-based recommendations from the Intergovernmental Panel on Climate Change, the United Nations body for assessing the science related to climate change.^3^ The short-term goal is to halve emissions by 2030, and institutions in HICs should make further and more rapid reductions. The United Nations–backed Race to Zero initiative provides strategic guidance and an accountability framework for reducing emissions in public and private sector organizations.^19^^,^^20^ Currently more than 5,000 businesses and 1,000 academic institutions have joined the Race to Zero. The Alliance for Sustainability Leadership in Education provides guidance for higher education institutions.^21^^,^^22^

For ASTMH, the ASTMH Green Task Force recommends building on the Society’s Green Statement (Box 2) by including an ambitious decarbonization goal in the next strategic plan. We also recommend including members with expertise in sustainability on the Board of Directors and including young people from both HIC and LMIC settings to help develop the implementation plan and deliver on the goal. ASTMH has a unique opportunity and a positive role to play by providing a platform for ASTMH members to learn from one another in terms of decarbonization and carbon literacy.

Working in global health often involves international travel both for field activities and sharing of scientific results. We need to approach travel in a way that is mindful of the greenhouse gas emissions it generates while recognizing that travel can be essential to building global health partnerships and performing needed research.^23^ In addition, many societies depend on in-person annual meetings for financial viability. We should prioritize virtual meetings and, when travel is necessary, opt for low-carbon means of transport (e.g., trains and electric vehicles as opposed to planes and vehicles with internal combustion engines). When flying is necessary, we can minimize emissions by flying direct, choosing economy class, and bundling meetings to reduce the frequency of flying. Finally, it is possible to offset the greenhouse gas emissions generated from air travel by capturing and storing greenhouse gases that would not otherwise be captured—for example, through reforestation. Online tools exist that calculate the amount of carbon dioxide equivalents emitted from a given flight and the number of trees needed to absorb the same amount. However, it takes approximately 20 years of tree growth to absorb the emissions.^15^

Most electricity is generated from a mix of renewable and fossil fuel sources. Institutions can switch to electricity suppliers that link directly to renewable energy generators (e.g., wind turbines), if available, or install their own renewable energy systems (e.g., solar panels and battery storage). Over time, gas heating systems can be replaced by electric systems that can be powered with renewable energy sources.

Laboratories consume significant amounts of energy, water, and other resources. The Laboratory Efficiency Assessment Framework (LEAF) and My Green Laboratory platforms offer evidence-based guidance and online frameworks for laboratory personnel and researchers to implement actions that limit waste and reduce the carbon footprint of laboratory work, such as switching ultra-low temperature freezers from –80 to –70°C.^24^^–^^26^ It is estimated that the LEAF platform saves on average the equivalent of 2.9 tons of CO_2_ per laboratory per year.^27^

Institutions may have investments in fossil fuels—for example, through holdings, endowments, or pension funds. The number of universities selling these investments and divesting from coal, oil, and gas is growing.^28^^–^^30^ Divesting from fossil fuels would align with the goal of global health institutions to improve global health.

A study of the decarbonization initiatives of 10 European global health institutions found that engaging internal stakeholders and collaboration with partners were important factors that facilitated progress.^31^ The ASTMH Green Task Force proposes that climate action within ASTMH and across the global health community can accelerate progress toward a healthier, more environmentally sustainable, and more equitable world.

Box 1 **The impacts of climate change worsen existing inequalities**

- Health-related risks are greater for those already vulnerable, such as children, pregnant women, the immunocompromised, and the elderly.
- Extreme weather affects different geographies of the world disproportionately.
- Poorer groups and people living in low and lower middle-income countries are at higher risk of climate impacts.
- The disruption caused by extreme weather events is associated with increased violence against women, girls, and other vulnerable people.

Box 2 **The American Society of Tropical Medicine and Hygiene (ASTMH) Green Statement adopted in 2021^32^**

Global climate change directly and indirectly impacts the spread of infectious diseases and threatens the public health progress made since the founding of our society. ASTMH membership recognizes that our activities, both individual and collective, influence climate change and that we can leverage our strengths to be better stewards of our interconnected planet.

To successfully carry out our society’s goals, we commit to the following:

- Holding ourselves responsible as a community for our impact by reducing the environmental footprint of the Annual Meeting and the *American Journal of Tropical Medicine and Hygiene*
- Encouraging interdisciplinary research and partnerships to advance environmental sustainability within our global health efforts
- Raising awareness within our society’s reach
